# Innovative Payment Mechanisms for High-Cost Medical Devices in Latin America: Experience in Designing Outcome Protection Programs in the Region

**DOI:** 10.3390/jmahp13030039

**Published:** 2025-08-04

**Authors:** Daniela Paredes-Fernández, Juan Valencia-Zapata

**Affiliations:** 1 Health Economics & Reimbursement, Medtronic South Latam, 532 Rosario Norte Street, Floor 12, Las Condes, Santiago 7561185, Metropolitan Region, Chile; 2 Health Economics, Policy, and Reimbursement, Medtronic Latin America, 701 Waterford Way, Suite 190, Miami, FL 33126, USA

**Keywords:** health financing and managed entry schemes, high-cost technologies, risk-sharing agreements, medical devices, cardiovascular

## Abstract

**Introduction and Objectives**: Risk-sharing agreements (RSAs) have emerged as a key strategy for financing high-cost medical technologies while ensuring financial sustainability. These payment mechanisms mitigate clinical and financial uncertainties, optimizing pricing and reimbursement decisions. Despite their widespread adoption globally, Latin America has reported limited implementation, particularly for high-cost medical devices. This study aims to share insights from designing RSAs in the form of Outcome Protection Programs (OPPs) for medical devices in Latin America from the perspective of a medical devices company. **Methods**: The report follows a structured approach, defining key OPP dimensions: payment base, access criteria, pricing schemes, risk assessment, and performance incentives. Risks were categorized as financial, clinical, and operational. The framework applied principles from prior models, emphasizing negotiation, program design, implementation, and evaluation. A multidisciplinary task force analyzed patient needs, provider motivations, and payer constraints to ensure alignment with health system priorities. **Results**: Over two semesters, a panel of seven experts from the manufacturer designed *n* = 105 innovative payment programs implemented in Argentina (*n* = 7), Brazil (*n* = 7), Colombia (*n* = 75), Mexico (*n* = 9), Panama (*n* = 4), and Puerto Rico (*n* = 3). The programs targeted eight high-burden conditions, including Coronary Artery Disease, atrial fibrillation, Heart Failure, and post-implantation arrhythmias, among others. Private providers accounted for 80% of experiences. Challenges include clinical inertia and operational complexities, necessitating structured training and monitoring mechanisms. **Conclusions**: Outcome Protection Programs offer a viable and practical risk-sharing approach to financing high-cost medical devices in Latin America. Their implementation requires careful stakeholder alignment, clear eligibility criteria and endpoints, and robust monitoring frameworks. These findings contribute to the ongoing dialogue on sustainable healthcare financing, emphasizing the need for tailored approaches in resource-constrained settings.

## 1. Introduction

Payment mechanisms in healthcare are the instruments insurers and payers use to purchase healthcare services on behalf of their beneficiaries [1]. Their complexity is described as a continuum, varying from fee-for-service systems to more complex pay-for-performance models [2,3]. In the search for valuable and innovative mechanisms for financing high-cost technologies, risk-sharing agreements (RSAs) have emerged as strategies to provide coverage with financial sustainability [4]. These are generally implemented with single or multiple objectives to mitigate clinical and financial uncertainties [5,6]. These agreements are especially valuable in risk-averse environments, where post-launch uncertainty around real-world performance and utilization can hinder optimal pricing and reimbursement decisions [7,8,9,10]. By linking payment to actual outcomes and managing both clinical and financial variability, RSAs represent a significant evolution in healthcare payment models, aligning incentives between payers and manufacturers while promoting more sustainable access to innovation. Most risk-sharing agreements (RSAs) in the European Union aim to control healthcare spending [11]. They are particularly useful when new medical technologies present uncertainties about their value and when payers must operate within limited and rigid budgets. RSAs also help when the actual number of patients using the technology exceeds initial estimates or differs from the population targeted initially in the product’s approval [12,13]. These functions underscore the flexibility of RSAs in adapting to real-world conditions while protecting payer budgets.

Risk-sharing models also help to control off-label prescribing and regulate prescriptions in populations or subgroups where the cost-effectiveness ratio is less favorable, preventing certain technologies from being prescribed for indications other than those for which they have been approved to avoid undesirable outcomes [12,14,15]. Although their implementation has been widespread globally, the same execution level is not documented in Latin America. Additionally, the experience, primarily in the form of risk-sharing agreements, has been predominantly applied to high-cost medications in oncological indications [11], with less emphasis on addressing high-cost medical devices. The latter is probably due to some of the reasons explored by Boriani, G. et al. (2022), such as “the characteristics of reimbursement practices and, in some cases, lack of consensus between administrators and physicians” [16]. Overall, these barriers underscore the need for tailored strategies to extend the benefits of risk-sharing agreements to a broader range of health technologies in the Latin American context.

There is significant interest in their utility in Latin America; however, there is a lack of systematic experiences from which to draw insights. This article aims to share the experience of designing risk-sharing agreements in the form of Outcome Protection Programs (OPP) for medical devices in Latin American countries from the perspective of a medical devices company.

## 2. Methods

These payment systems follow the general framework of traditional payment mechanisms, involving a trade-off of financial risk but introducing new variables for pricing [4,14]. This evolution reflects a shift towards more sophisticated models that seek to balance innovation with financial accountability.

The experience of designing innovative financing mechanisms focuses on identifying their classic dimensions: payment base, access criteria, price, risks, pricing, and performance incentives, whether clinical or financial [14,17]. These variables will involve a set of specific parameters. Understanding these foundational components is essential for constructing robust and transparent risk-sharing agreements.

The payment base includes the services (medical care, medical technology, follow-up, and education, among others) covered by the innovative payment mechanism. The access criteria consist of rules ordering patient entry into the scheme, which may be demographic, clinical, or requirements to achieve the desired outcome [14]. The definition of these parameters should not be discretionary. All definitions related to sociodemographic profiles, clinical criteria, and other requirements must be based on evidence and recommendations to achieve the best patient outcomes [4]. The most transparent and equitable way to express these definitions is to consider each therapy’s eligibility and exclusion criteria according to subgroups. Establishing evidence-based and subgroup-specific parameters ensures fairness and clinical coherence in the design of these financial instruments.

On the other hand, risks can be categorized into three types: financial, clinical, and operational. The first type of risk is related to a more significant budgetary impact due to uncontrolled variables such as a higher number of patients than anticipated, higher rates of resource use or consumption, or other phenomena resulting from failures in identifying the payment base and access criteria. Clinical risk refers to those experienced by patients, which can range from complications to supply-induced demand or even iatrogenic effects. Finally, operational risk is faced by the organization providing services and includes managing higher patient flows, overloaded processes, or increased transaction costs [18]. The latter, for example, refers to the additional workload generated by managing the innovative financing model, such as the need to enter additional clinical data into a patient record platform for monitoring purposes [4]. Understanding and managing these distinct categories of risk is essential to the effective and sustainable implementation of innovative payment models.

In terms of incentive mechanisms, innovative financing programs must include performance metrics. Some metrics explored in the evidence include survival, mortality, and surgical reinterventions [5]. These schemes must include reimbursement configurations that compensate providers for meeting those metrics. This configuration must be explicitly connected to the pricing scheme. The pricing scheme results from combining rules to establish a payment base, access criteria, and risk control [14]. By aligning incentives with measurable outcomes, these programs promote accountability and support value-based healthcare delivery.

Our experience incorporates recommendations from the model Paredes, D., and Lenz, R. (2019) [4] proposed for RSA in Latin America. This model suggests that the innovative financing initiative should be framed within an opportunity window, meaning a context of need that enables negotiation and the willingness of stakeholders to engage in the design, implementation, and evaluation process. The design phase emerges after, subdivided into activities specific to the entity providing the technology and activities arising from the program negotiation between the technology producer and the provider or payer [4]. This framework underscores the importance of timing and stakeholder readiness as prerequisites for successful agreement development. The model for risk-sharing agreements proposed by Paredes, D., and Lenz, R. (2019) [4] in the Latin American context was adapted in this experience by streamlining the sequence of implementation stages and placing particular emphasis on the early formation of a dedicated task force. A key modification involved the architectural design of the agreement. Instead of relying on a fully centralized model construction process, the initial proposal is developed by the provider and subsequently calibrated in collaboration with the payer. This approach enhances efficiency in the model-building phase, facilitates technical alignment from the outset, and fosters greater ownership by the provider—factors that have proven critical for timely implementation in resource-constrained environments. By integrating these adaptations, the model becomes more responsive to real-world constraints while preserving its strategic foundation.

Typically, as a result of an internal work by the producer, initiatives begin with the identification and characterization of a pathology, followed by defining the dimensions of the payment base, access criteria, risks and their control mechanisms, and the pricing scheme with the definition of success metrics [19]. This initial proposal is refined in a more in-depth negotiation stage between the stakeholders. In this stage, the initial definitions are fine-tuned. Also, parameters such as the responsibilities of each party at each stage of the innovative mechanism, the time horizon of the agreement, exit clauses, arbitration, the frequency of performance metrics measurement, and the management of information generated by the agreement are defined [4]. This approach ensures that both technical soundness and mutual alignment are embedded from the outset, increasing the likelihood of successful implementation.

In identifying endpoints of interest, or as referred to in this experience, admissible events, it is critical to ensure the availability of easy verification methods to report and analyze. Additionally, programs should ensure the effective capability for longitudinal and serial monitoring of the endpoints within the patient cohort. Finally, programs must consider all these elements and determine whether the metric-based pricing will be conducted at the individual patient level or the cohort level. This will be determined by the analytical capacity of the teams and the associated transaction costs, such as those for notification, billing, and others. These considerations collectively ensure that endpoint measurement is both feasible and aligned with operational capacities, thereby supporting accurate and sustainable financing models.

In our experience, the selection of program parameters varies depending on the specific dyad of therapy and disease and the context of the potential implementing institution or payer. Elements such as expected surgical or clinical event volume, learning curves, and organizational characteristics are considered. Clinical parameters are derived from a review of the published clinical evidence, using reported endpoints that are then adapted to real-world conditions through a process to translate efficacy into efficiency and performance. Depending on data availability within each institution, these parameters may be further calibrated using real-world data, interim analyses, or institutional reports. Each contract model undergoes a final adjustment process to ensure the adopting organization is comfortable with the selected parameters. This iterative and context-sensitive approach helps align program design with practical realities, improving the likelihood of successful implementation and meaningful outcomes.

**Figure 1:**
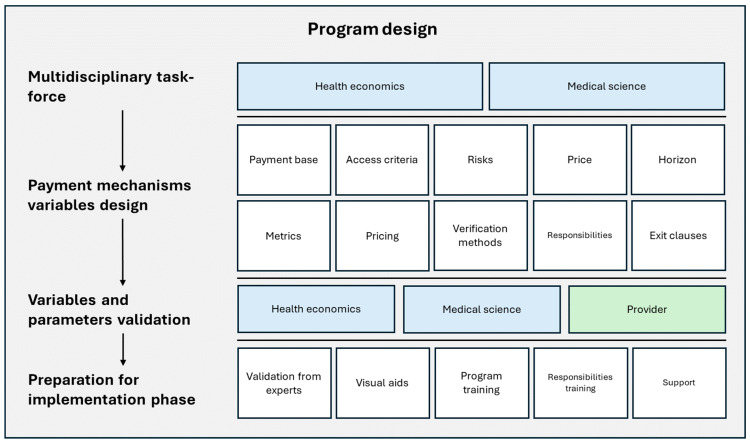
General Model for Designing risk-sharing agreements in the Form of Outcome Protection Programs for High-Cost Medical Devices. Source: Model adapted by the authors from Paredes, D., Lenz, R., 2019. Proposed Model of risk-sharing agreements.

Figure 1 summarizes the experience referred to as the General Model for Designing risk-sharing agreements in the Form of Outcome Protection Programs for High-Cost Medical Devices. The process begins with the formation of a multidisciplinary task force, bringing together two core technical areas: health economics and medical science.

The first step involves building the foundational structure of the contractual model by defining key payment mechanism elements. These include the payment basis, access criteria, risk-sharing structure, pricing, time horizons, performance metrics, verification methods, responsibilities, and contract exit clauses. Next, the variables and parameters of the agreement are defined and validated. This requires close collaboration between the technical teams and the healthcare provider to ensure that all contractual terms are fair and meaningful to both parties. Following this, a preparation phase for implementation is undertaken. During this stage, the contract architecture is reviewed and validated by external experts, and visual aids are developed to assist clinical teams in identifying eligible events and facilitating timely reporting. Training is also provided to ensure proper execution of the agreement, including an understanding of the innovative payment model, the performance metrics, access and eligibility criteria, and the procedures for reporting outcome-related events. This collaborative and preparatory approach ensures that all stakeholders are equipped with the knowledge and tools necessary for successful contract execution. Importantly, this phase also includes medical education activities aimed at promoting the rational use of the technology and ensuring its appropriate indication within eligible patient subgroups. Thus, the integration of education and training further strengthens the implementation process, enhancing both clinical and economic success.

For our experience, a Medtronic task force of seven health economics and medical sciences experts designed the innovative payment mechanisms over two semesters for a Latin American context. These experiences were developed in Argentina, Brazil, Colombia, Mexico, Panama, and Puerto Rico. The experience covers the period from 2021 to early 2025, during which the team has continuously developed and implemented outcome protection programs (OPPs), now expanding into areas such as diabetes and spine surgery.

Regarding the opportunity window, it has been pertinent to explore pathologies and intervention needs among stakeholders where there is an urgent demand for innovative models. This was combined with prevalence and incidence factors at the Latin American level, the provider’s capacity to meet demand, and the experience of the involved stakeholders in managing these programs. In this experience, therapies focused on interventional management in hemodynamic laboratories across Latin America have been prioritized for the following eight conditions: i. Coronary Artery Disease, ii. Peripheral Arterial Disease of the Lower Limbs, iii. Abdominal Aortic Aneurysm, iv. Heart Failure, v. Skin Pocket Infection associated with implantable devices, vi. Paroxysmal or Persistent Atrial Fibrillation, vii. Inappropriate medical device shock, and viii. Post-implantation Cardiac Rhythm Disorders associated with Percutaneous Aortic Valve Replacement. The counterpart in this experience has been predominantly private providers (80%) and payers in the region (20%).

The following assessment framework summarizes the needs and motivations specific to the demand segment (patients) and the supply segment (technology producers and providers) within the opportunity window.

**Figure 2:**
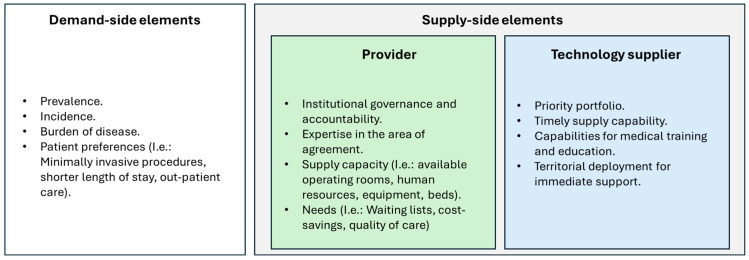
Framework for the assessment of needs and motivations for program prioritization. Source: Prepared by the authors.

Figure 2 outlines the Framework for the Assessment of Needs and Motivations for Program Prioritization. On the demand side, the analysis focused on identifying whether a meaningful opportunity existed for implementation. This involved assessing disease prevalence and incidence, the burden of disease, and patient adherence preferences—elements that help determine both the scale and urgency of the need, as well as the potential for health impact. This demand-side evaluation is crucial for prioritizing programs that address the most pressing healthcare challenges effectively. On the supply side, the assessment was divided into two key stakeholders: healthcare providers and technology suppliers.

For healthcare providers, the evaluation emphasized institutional governance and accountability as foundational elements to determine their readiness to implement innovative contractual models. This included assessing whether providers could ensure proper organizational adherence to eligibility criteria and timely case reporting. In addition, providers’ clinical expertise was considered—particularly their specialization in the disease area covered by the proposed contract—under the rationale that institutional strength in the relevant clinical domain would support optimal performance. Providers were also assessed on their operational capacity to absorb new demand, using indicators such as the availability of surgical theaters, adequacy of human resources, equipment availability, and bed capacity. Finally, their institutional needs were examined—such as challenges with waiting lists, cost-containment objectives, or quality improvement goals—to determine whether these needs created a favorable context for implementing the proposed model. Together, these criteria allow for a comprehensive appraisal of provider readiness, ensuring successful adoption of innovative payment models. For the technology supplier, the assessment considered whether the prioritized therapy aligned with the supplier’s strategic portfolio. Additional factors included their ability to ensure a timely and uninterrupted supply of the technology, their capacity to provide medical training and clinical education as a quality-enhancement strategy, and their operational footprint—specifically, the ability to deploy local teams for immediate on-site support when required. This thorough supply-side evaluation ensures that the technology supplier can effectively support the program’s operational and clinical demands.

The role of these programs was recognized for aligning providers with evidence-based indications and eligibility criteria. This alignment results in benefits by limiting variance in both outcomes and costs. The latter point is particularly critical from the perspective of funders or health insurers, who view these programs as systems for mitigating expenditure uncertainty. However, while risk-sharing agreements and outcome protection programs stipulate the eligibility criteria that will govern the scheme, institutional governance elements are crucial for aligning teams with the program’s parameters and variables [4]. Strong institutional governance ensures that these programs function effectively, promoting both clinical adherence and financial predictability.

## 3. Results

Table 1 outlines the principal dimensions and parameters of the designed *n* = 105 programs. We have applied the taxonomy proposed by Ferrario, A. and Kanavos, P., from the London School of Economics and Political Science, LSE publication (2013), and ISPOR [5].

Access criteria have been set according to the primary therapy endpoint, as these payment mechanisms account for these metrics in real-world settings. For each disease and technology, we have defined access criteria for the operation of the OPP. Also, the type of RSA and OPP to develop must be practical and administratively straightforward for both parties. This implies the examination of commercial procedures as well as determining the best course of action, considering a smooth operation.

The first cohort of OPP programs in Latin America was developed for coronary disease, Abdominal Aortic Aneurysm, peripheral vascular disease, and cardiac implantable electronic device (CIED) infection (Table 1 Part A).

In a second period, four additional programs were designed, including Heart Failure, inappropriate shock reported in patients using cardiac resynchronization with defibrillator devices, Paroxysmal Atrial Fibrillation or Persistent Atrial Fibrillation, and post-transcatheter aortic valve replacement (TAVR) arrhythmia. Including both cohorts, 11.4% (*n* = 12) OPP programs were designed for post-TAVR arrhythmia, and this OPP resulted in the most interest for providers during the second cohort. Remarkably, this program included providing a transvenous pacemaker if arrhythmias were reported within the first month after a TAVR replacement was implanted using manufacturer recommendations (cusp overlap technique). The program would supply as many pacemakers as needed until reaching a financial cap agreed upon between both parties, based on clinical evidence and adjusted by learning curves (Table 1 Part B).

The specification of financial caps in some programs incentivizes clinical providers to adhere to best clinical practices and manage clinical risks within evidence-based limits. To establish these caps, reference trials were analyzed, and financial thresholds were set based on their metrics. Financial caps are calculated based on evidence regarding the occurrence of the admissible event. The rationale is that the number of events should not be expected to exceed those reported in the available evidence. The tolerance margin for any excess in events reported by the provider—relative to the evidence—is a parameter that is calibrated during the contract negotiation. Financial caps also act as a signal for providers to mitigate risks and align medical staff with quality metrics. Once the financial cap is reached, the OPP program is suspended until the next renewal period. Based on our experience, financial caps have accounted for learning curves, as outcomes observed in clinical trials may differ during the early stages of implementation. This phenomenon is especially relevant in medical devices in surgical care [20] and cardiovascular procedures [21], and has been studied in Transcatheter Aortic Valve Replacements [22]. This adjustment must be negotiated between both parties.

Finally, the experience has included systematic monitoring of model adoption per country, tracking the occurrence of admissible events by provider, and examining the underlying causes of each one. This approach aims to support continuous quality improvement initiatives at the provider level. These programs have reinforced clinical governance by aligning clinical teams around standardized eligibility criteria and outcome metrics, reducing variability and improving results. By transferring part of the financial risk to technology suppliers, they also enhance financial protection and cost predictability for providers. For patients, the model promotes safer, more effective care. More broadly, OPPs help align clinical and managerial teams toward common goals, supporting both better outcomes and the financial sustainability of health systems.

**Figure 3:**
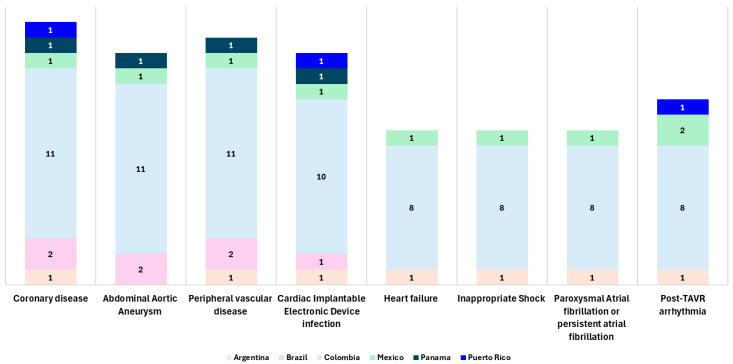
Geographic distribution of Outcome Protection Programs for High-Cost Medtronic Medical Devices in Latin America, sorted by disease or health condition.

Countries adopting these models vary by disease and therapy. Colombia led 71.4% of contracts (*n* = 75). Eleven of them for coronary disease requiring Drug-Eluting Stents; another eleven contracts for Abdominal Aortic Aneurysm treated with aortic endoprosthesis; and the same amount for peripheral vascular disease treated with a drug-coated balloon. In second place, Mexico led 8.6% (*n* = 9) of OPP programs. In Latin America, a total of 16.2% (*n* = 17) OPP programs were designed for coronary disease, 15.2% (*n* = 16) for peripheral vascular disease, 14.3% (*n* = 15) for Abdominal Aortic Aneurysm, and also 14.3% (*n* = 15) for cardiac implantable electronic device (CIED) infection (Figure 3).

As potential enablers, in the case of Colombia, the Ministry of Health enacted the Law “Decreto 441 de 2022” to facilitate negotiations among stakeholders, including clinics, hospitals, and insurance companies. This legislation introduced various payment models and emphasized the implementation of outcome measures in discussions between healthcare providers and insurers. As a result of this agreement, the Colombian healthcare system began to adopt various negotiation modalities, notably including risk-sharing agreements. These innovative approaches aimed to align incentives across different parties, ensuring that all stakeholders worked towards shared goals of improving patient outcomes and managing costs effectively. By pioneering the implementation of these models, Colombia’s healthcare system demonstrated exceptional performance, highlighting its commitment to advancing healthcare delivery and efficiency. This proactive approach fostered a collaborative environment and set a benchmark for other countries in the region, showcasing the potential benefits of structured negotiation frameworks in healthcare.

## 4. Discussion

In Latin America, various entities have deployed efforts to establish value frameworks to facilitate consensus for establishing these initiatives. One such effort is the Value-Based Health Learning Community of the Institute of Clinical Effectiveness and Health (IECS) in Argentina, which, in its role as a neutral entity, brought together payers, providers, and industry [23]. The work of Paredes, D., and Lenz, R. (2019) highlighted the importance of the neutral entity’s role in enabling the design and implementation phases [4]. Additionally, the experience in Latin America highlights the need to include specialized health institutions with well-defined treatment protocols for the pathology subject to these experiences [24]. The same experience underscores the importance of the negotiation skills of the parties and technical expertise [24]. Towse, A. and Fenwick, E. (2024) recommend that risk-sharing agreements should be practical, so they become an option for payers [25]. Ultimately, the success of innovative payment models in Latin America depends on collaborative frameworks, technical competence, and pragmatic approaches that address real-world challenges.

In the Chilean experience led by the ministerial entity, the work of Poblete, S. (2020) examines the profile of different types of risk-sharing agreements proposed by the industry—primarily for molecules. Among the schemes, the most common were unit-based rebates, portfolio discounts, and expenditure caps [26]. Similarly, much of the Chilean experience in pharmaceuticals has proposed innovative reimbursement mechanisms for breast cancer, such as dose limits and portfolio discounts, with a strong emphasis on survival metrics as parameters for pricing [27,28].

A systematic review examined performance-based risk-sharing arrangements in the United States. In almost two decades, out of *n* = 52 schemes for diagnostics and devices, only 12,4% corresponded to programs for cardiology interventions [29]. The same experience highlights the greater focus of private providers on developing risk-sharing models for medical devices [29]. In the United States, Medtronic implemented the most extensive OPP program for infections related to cardiac implantable electronic devices using the TYRX™ absorbable antibacterial envelope. From January 2017 to August 2021, out of 1568 sites with OPP programs ongoing, infection rates dropped to 0.15% compared to 0.7% observed in the WRAP-IT trial. After 92,044 surgeries, 141 rebates were paid to those institutions reporting infections despite using the envelope. This is a return of USD 1,410,000 for the period. The authors highlight that treating the estimated additional infections that could have occurred without the use of TYRX™ would have led to approximately USD 23.6 million in additional costs for the program sites [30].

One parameter often overlooked in RSA initiatives is the determination of time horizons. This should consider two effects: the time needed to capture benefits and the medical technology’s innovation velocity. The first relates to a reasonable period from the perspective of the signatories to capture the desired benefits, that is, horizons that allow for the expression of admissible events or endpoints, and additionally, a period that accounts for the speed of innovation of medical devices. Properly defining time horizons is crucial to ensure that agreements are both effective and adaptable to real-world clinical and technological changes. Although not in medical devices, one of the notable failures in determining appropriate time horizons in high-cost reimbursement systems is the case of beta-interferon for multiple sclerosis. This RSA involved a clinical trial and a ten-year contract [31] without considering the appearance of lower-cost alternatives in the short term without exit clauses [12]. This example highlights the risks of inflexible contracts that fail to adapt to evolving market conditions and technological advancements. Therefore, this experience has considered time horizons of up to three years to provide flexibility to the parties, allow sufficient time for the expression of any endpoint or admissible event related to access criteria, and facilitate the incorporation of potentially new technologies with a better economic profile if needed. Such flexible time horizons promote sustainability and responsiveness in innovative payment models, enhancing their overall success.

Our manuscript has reported the design experience of these models in Latin America. Although reviewing the implementation of this experience is beyond the scope of this work, we have learned that experiences should include a preparation period for the implementers. Clinical inertia is a phenomenon observed in this experience, referred to as the tendency to maintain existing clinical and administrative practices before the signing of the program, and difficulties in incorporating new tasks entailed by the contract. This should be addressed with multidisciplinary training sessions, ensuring that all necessary teams are familiar with the details, associated claim forms, expected outcomes, and endpoints of interest, reinforcing each participant’s tasks, enhanced with visual aids placed in high-traffic areas for clinical and administrative teams. Effective preparation and training are essential to overcome resistance and ensure smooth adoption of innovative payment models.

Also, problems in identifying admissible events by the supply side may be rooted in the multiplicity of financing systems to manage, leading to practical difficulties that implementers face in notifying events and managing accumulated risk, as discussed in the experience of pharmaceuticals [32,33]. Whilst each financing experience limits risks and deploys control tools, the cumulative effect of all innovative and traditional financing systems results in a greater risk to manage. Zaric, G. (2021) alerts on undesirable effects of risk-sharing models that should be considered in negotiations, including payers, manufacturers, and providers [34]. Recognizing and addressing the compounded complexity of multiple financing systems is vital for sustainable and effective risk-sharing agreements.

Risk-sharing agreements have been promoted to gain more knowledge about the clinical effectiveness of technologies, manage their use to optimize performance, ensure that the funded therapy delivers the results promised by the manufacturer, and make estimates of its health impacts. Additionally, they aim to understand the practical implications associated with their use [11,13,15]. These agreements serve as vital tools for aligning clinical outcomes with real-world effectiveness and accountability in healthcare financing. It is proposed that while each experience increases the level of knowledge, it is much more effective when experiences are studied together rather than in isolation. The synergy of experiences, whether through an integrated system of regional registries or other mechanisms, allows for the accumulation of evidence and can even enhance the statistical power of results. Furthermore, when risk-sharing agreements are studied collectively, the likelihood of duplicating efforts is reduced, and international experience can be leveraged to navigate local complexities [11]. Additionally, according to Parkinson, M. et al. (2019), manufacturers of medical devices should consider this kind of payment model as part of their strategies for gaining access [35] while ensuring sustainable models to ensure the quality of care. Collective analysis and cooperation enhance the impact and sustainability of these agreements, benefiting all stakeholders involved.

This study is subject to several important limitations. The limited availability of empirical data and the relatively nascent application of risk-sharing agreements (RSA) to medical devices constrain the scope and depth of the analysis, requiring reliance on the more established experience with pharmaceuticals. While the use of interim expert opinion was necessary to inform assumptions and contextual interpretation, it carries an inherent risk of selection bias as the perspectives of a limited group of stakeholders may not fully represent the diverse views across the healthcare ecosystem, despite careful efforts to ensure a balanced and relevant group of participants. Additionally, this study reflects the experience of a specific region, which may limit the generalizability of the findings, as our experience may not be directly generalizable to other health systems, therapeutic areas, or technologies. These limitations underscore the need for further empirical research and real-world evidence to validate and refine the insights presented.

The prioritization of high-burden diseases and the use of evidence-based eligibility criteria, while effective in promoting value-based healthcare delivery, may also restrict the applicability of Outcome Protection Programs (OPPs) to other pathologies or conditions that were not included in the scope of this study. This narrow focus may limit the broader applicability of RSAs to diverse healthcare needs. The study primarily reflects experiences from private healthcare providers, which may not accurately capture the complexities and constraints faced by public healthcare systems. The differences in governance structures, regulatory frameworks, and social security models across countries further limit the applicability of the results to other settings.

Most of the documented experiences in our report were developed in collaboration with private healthcare providers, likely due to their greater contractual flexibility to engage in innovative payment models. As mentioned above, consequently, the implementation of such contracts in the public sector may differ, as it must account for the specific legal and regulatory frameworks applicable in each country. Therefore, context-specific adaptations and careful consideration of regulatory environments are essential for successful implementation across diverse healthcare systems. For instance, in Peru, the recent Public Procurement Law (Law No. 32069) introduces mechanisms that may enable innovative contracting [36], while in Chile, Exempt Resolution No. 410 (2025) outlines updated guidelines that may support such arrangements within the public sector [37]. Based on these new updates, public providers are incentivized to propose these models. These legal advances set the stage for a more flexible and innovation-friendly contracting environment in the region. Recent developments in the region have sought to actively promote legal and institutional frameworks that enable innovation within public sector and social security institutions. The experiences documented in this study offer pragmatic and operational insights into the design and implementation of these models, contributing to the development of local expertise and institutional learning. Such initiatives demonstrate how regulatory evolution can drive practical improvements and capacity building in healthcare financing. This accumulated knowledge may serve as a valuable foundation for future adaptation and scaling within national health systems.

To address these limitations, future research should aim to conduct longitudinal studies and collect comprehensive data on RSA implementations across diverse healthcare settings and conditions; it also should diversify stakeholder engagement involving a broader range of stakeholders, including public health officials, payers, patients, and providers, to capture diverse perspectives and enhance the robustness of findings. Future research also should improve generalizability by investigating how RSAs can be adapted to different healthcare systems, regulatory environments, and disease areas to promote scalability and applicability. Finally, future experiences should enhance methodological rigor by employing rigorous research methodologies, such as randomized controlled trials and real-world evidence studies, to validate RSA effectiveness and minimize reliance on expert opinion. By explicitly acknowledging these limitations and incorporating recommendations for future research, this study contributes to the ongoing dialogue on innovative healthcare financing mechanisms and their potential to improve outcomes in resource-constrained settings.

## 5. Conclusions

The experience of designing risk-sharing agreements in the form of Outcome Protection Programs for high-cost medical devices in Latin America highlights the potential of these innovative financing mechanisms to address clinical and financial uncertainties in resource-constrained settings. Such mechanisms have emerged as essential tools to balance the complex needs of healthcare systems while managing costs and outcomes effectively.

The structured design process, which includes defining payment bases, access criteria, risk mitigation measures, and success metrics, demonstrates that these agreements can effectively align the interests of patients, providers, payers, and manufacturers. In particular, the prioritization of high-prevalence pathologies with urgent demand and disease burden, combined with evidence-based eligibility criteria, facilitates value-based healthcare delivery while promoting budget predictability and expenditure control. This alignment is crucial for ensuring that all parties are incentivized towards optimal clinical and economic outcomes.

Successfully implementing risk-sharing agreements requires robust institutional governance, continuous training programs, and efficient systems for longitudinal monitoring of outcomes. Nevertheless, the regional heterogeneity in healthcare systems and the multiplicity of financing arrangements present significant challenges to the widespread adoption of these mechanisms, and each country needs to tailor OPP programs to their particular needs and managerial models. To overcome these challenges, fostering collaborative efforts among stakeholders and establishing regional registries to consolidate evidence will be essential to optimize the design and performance of OPP programs or any RSA experience, ultimately contributing to a more sustainable and equitable healthcare system in Latin America.

## Figures and Tables

**Table 1:** Main Characteristics of Outcome Protection Programs for High-Cost Medical Devices in Medtronic Latin America.

Part A. First Cohort of OPP Programs				
Disease	Coronary Disease	Abdominal Aortic Aneurysm	Peripheral Vascular Disease	Cardiac Implantable Electronic Device (CIED) Infection
**Medical device**	Resolute Onyx^TM^ Medtronic and subsequent generations of Drug-Eluting Stent	Aortic Endoprosthesis Endurant II/IIs^TM^ Medtronic	Drug-coated balloon In.Pact Admiral^TM^ Medtronic	Absorbable antibacterial envelope Tyrx^TM^ Medtronic
**RSA Taxonomy**	Outcome guarantee	Outcome guarantee combined with financial cap	Outcome guarantee	Outcome guarantee combined with financial cap
**Access criteria** **(admissible event)**	Reported cases with a Restenosis event in the same lesion site from day 30 to day 365 after the implant date	Needed reinterventions for an Abdominal Aortic Aneurysm within the next 2 years at the same site of primary lesion	Reported cases with restenosis at the same site as the initial lesion within one year after the angioplasty procedure	Reported infection at the implant site (skin pocket) during the first year after implant
**Guarantee (Input for pricing and incentives)**	The manufacturer will provide a Drug-Eluting Stent and/or coronary balloon per lesion as needed	The manufacturer will provide proximal cuffs, distal extension, or complete endoprosthesis as needed until a financial cap is reached	The manufacturer will provide a drug-coated balloon as needed	The manufacturer will provide an absorbable antibacterial envelope Tyrx^TM^ plus an equivalent Cardiac Implantable Electronic Device (CIED) until a financial cap is reached
**Countries where implemented**	Argentina (*n* = 1) Brazil (*n* = 2) Colombia (*n* = 11) Mexico (*n* = 1) Panama (*n* = 1) Puerto Rico (*n* = 1) Total *n* = 17 programs	Brazil (*n* = 2) Colombia (*n* = 11) Mexico (*n* = 1) Panama (*n* = 1) Total *n* = 15 programs	Argentina (*n* = 1) Brazil (*n* = 2) Colombia (*n* = 11) Mexico (*n* = 1) Panama (*n* = 1) Total *n* = 16 programs	Argentina (*n* = 1) Brazil (*n* = 1) Colombia (*n* = 10) Mexico (*n* = 1) Panama (*n* = 1) Puerto Rico (*n* = 1) Total *n* = 15 programs
**Part B. Second Cohort of OPP Programs**				
**Disease**	**Heart Failure**	**Inappropriate Shock**	**Paroxysmal Atrial** **Fibrillation or Persistent Atrial Fibrillation**	**Post-TAVR** **Arrhythmia**
**Medical device**	Cardiac Resynchronization Therapy with Defibrillator (CRT-D) with Adaptiv CRT^TM^ algorithm Medtronic	Cardiac Resynchronization Therapy with Defibrillator (CRT-D) or Implantable Cardioverter Defibrillator (ICD) with SmartShock^TM^ algorithm Medtronic	Cryoballoon Medtronic	Transcatheter Aortic Valve Replacement (TAVR) plus Pacemaker Medtronic
**RSA Taxonomy**	Money-back guarantee with financial cap	Money-back guarantee with financial cap	Money-back guarantee with financial cap	Outcomes guaranteed with financial cap
**Access criteria** **(admissible event)**	Cases reporting a Heart Failure hospitalization (readmission event) within 90 days post CRT-D implant with an AdaptivCRT™ algorithm	Cases hospitalized or visiting the emergency room due to an inappropriate shock after CRT-D or ICD implant with a SmartShock™ algorithm	Cases reporting atrial fibrillation needing hospitalization from day 90 to day 365 after an index Cryoballoon procedure	Cases needing a transvenous pacemaker from day 1 to 30 following a Transcatheter Aortic Valve Replacement using a Cusp Overlap Technique
**Guarantee (Input for pricing and incentives)**	The manufacturer will activate a negotiated rebate until a financial cap is reached	The manufacturer will activate a negotiated rebate until a financial cap is reached	The manufacturer will activate a negotiated rebate until a financial cap is reached	The manufacturer will provide a new transvenous pacemaker for free
**Countries where implemented**	Argentina (*n* = 1) Colombia (*n* = 8) Mexico (*n* = 1) Total *n* = 10 programs	Argentina (*n* = 1) Colombia (*n* = 8) Mexico (*n* = 1) Total *n* = 10 programs	Argentina (*n* = 1) Colombia (*n* = 8) Mexico (*n* = 1) Total *n* = 10 programs	Argentina (*n* = 1) Colombia (*n* = 8) Mexico (*n* = 2) Puerto Rico (*n* = 1) Total *n* = 12 programs

## Data Availability

More information upon requirement to corresponding author.
